# Wireless Sensor Network Deployment: Architecture, Objectives, and Methodologies

**DOI:** 10.3390/s25113442

**Published:** 2025-05-30

**Authors:** Frantz Tossa, Yves Faga, Wahabou Abdou, Eugène C. Ezin, Pierre Gouton

**Affiliations:** 1 LETIA Laboratory, University of Abomey-Calavi, Abomey-Calavi BP 2549, Benin; 2 ImViA Laboratory, University of Bourgogne Franche-Comté, 21000 Dijon, France

**Keywords:** wireless sensor network (WSN), sensor architecture, deployment method, optimization, detection models

## Abstract

Wireless Sensor Networks (WSNs) have become a critical technology in the Internet of Things (IoT) era, enabling the seamless integration of sensing, communication, and computational capabilities. WSNs have emerged as a transformative technology with applications ranging from environmental monitoring to industrial automation. As WSNs become more prevalent in various applications, the deployment phase takes center stage in defining their efficiency, effectiveness, and scalability. This paper provides a comprehensive overview of the multifaceted landscape of WSN deployment strategies. Digging deeper into sensor nodes, deployment types, goals, sensing patterns, and deployment methodologies, it comprehensively explores the multifaceted domain of WSN deployment and provides a map for optimizing the deployment across a spectrum of applications.

## 1. Introduction

Wireless Sensor Networks (WSNs) have emerged as a cornerstone technology in modern cyber-physical systems, enabling real-time monitoring, environmental sensing, and intelligent decision-making across diverse domains including environmental monitoring, healthcare, industrial automation, and smart cities [1]. Comprised of spatially distributed sensor nodes equipped with sensing, processing, and wireless communication capabilities, WSNs facilitate large-scale, autonomous data acquisition and transmission. Historically, the origin of sensor networks can be traced back to the Cold War, with the deployment of acoustic arrays in the Sound Surveillance System (SOSUS) to detect Soviet submarines [2]. Since then, advances in microelectronics, low-power communications, and embedded computing have fueled the proliferation of WSNs, making them integral to both civilian and military applications.

Several studies have been conducted on the deployment of WSNs [3,4,5,6]. Despite their promise, the deployment of sensor nodes remains one of the most critical and complex challenges in the design of WSNs. Sensor nodes differ in terms of energy sources, processing capabilities, communication ranges, and sensing modalities. This heterogeneity, while enabling application-specific customization, complicates deployment strategies. Moreover, the requirements for deployment vary dramatically between applications: some demand full area coverage, others emphasize connectivity, fault tolerance, minimal energy consumption, or real-time responsiveness. As such, deployment must reconcile competing objectives, including coverage optimization, network lifetime maximization, cost efficiency, and robustness to environmental dynamics.

Although several surveys have reviewed aspects of WSN deployment, most focus narrowly on either coverage techniques, energy-efficient routing, or specific algorithmic approaches. For example, some reviews examine node placement optimization algorithms without considering broader architectural and sensing models, while others focus on clustering strategies or mobile node positioning with limited generalizability. These fragmented perspectives fail to provide a unified view of the design space that integrates architecture, objectives, sensing models, and deployment methodologies.

The key contribution of this paper is to offer a comprehensive, structured, and critical overview of WSN deployment strategies, distinguishing it from prior surveys. Specifically, this paper does the following:

1. Reviews the general architecture of sensor nodes, identifying how hardware and software characteristics affect deployment strategies.
2. Categorizes deployment objectives and aligns them with practical application scenarios.
3. Compares sensing models and explains their role in defining coverage constraints.
4. Classifies deployment methodologies (deterministic, random, hybrid), highlighting their trade-offs, constraints, and suitability.
5. Critically analyzes the strengths and limitations of current techniques.
6. Identifies open research challenges and proposes future research directions for more intelligent, adaptive, and resilient deployments.

By synthesizing architectural aspects, sensing paradigms, and optimization strategies under a unified framework, this survey aims to provide both novice researchers and seasoned practitioners with a deeper and broader understanding of WSN deployment. In doing so, it bridges gaps left by earlier surveys and sets the stage for the development of next-generation WSN deployment solutions tailored to increasingly dynamic and heterogeneous environments.

This paper is organized as follows. Following this introduction, Section 2 describes sensor nodes, their components, and node types. In Section 3, deployment types are presented. Section 4 discusses the main goals that can be achieved during deployment. Section 5 and Section 6 are devoted, respectively, to the different detection models and deployment methodologies, and precede the conclusion.

## 2. Sensor Nodes

### 2.1. Components of a Sensor Node

Depending on the application, there are several types of sensors. However, they remain, for the most part, with a similar hardware architecture. A sensor is mainly composed of a unit: capture (acquisition), processing, communication, and energy. Additional components such as a GPS (Global Positioning System), a mobility system or an energy generator [7,8] can be added depending on the area of application.

- The capture unit is composed of two sub-units, including a physical capture device that takes information from the local environment and an analog/digital converter called ADC (Analog to Digital Converter). The sensor is responsible for providing analog signals that the ADC transforms into a digital signal understandable by the processing unit.
- The processing unit is generally a processor coupled to a random access memory. Its role is to control the proper functioning of the other units. On some sensors, it can embed an operating system to operate the sensor. It can also be coupled to a storage unit, which will be used to record the information transmitted by the capture unit. This unit can also be equipped with an intelligence enabling it to analyze/process the data acquired by the capture unit.
- The communication unit performs all transmissions and receptions of data on a wireless medium. It can be of the optical type or of the radio-frequency type.
- The unit of energy, usually a battery of small size and limited capacity. This often makes energy a sensor’s most valuable resource, as it directly affects its lifespan.

**Figure 1:**
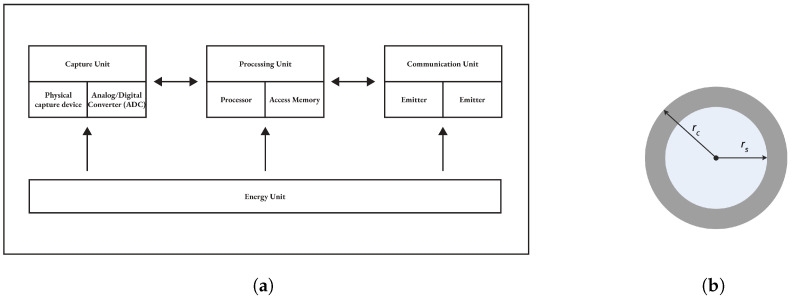
Components of a sensor node. (**a**) Architecture of a sensor node. (**b**) Sensing and communication range.

The acquisition unit is the physical part that makes it possible to take the measurement, while the communication unit is responsible for disseminating the data. Each sensor node has a communication range ($r_{c}$) and a detection or sensing range ($r_{s}$). The communication range $r_{c}$ is the maximum distance at which the sensor node can communicate with other nodes, while the detection range $r_{s}$ is the maximum distance around which the sensor node can detect the event [7,9]. Figure 1a illustrates the architecture of a sensor node, while Figure 1b shows the areas defined by the two scopes.

### 2.2. Types of Sensor Nodes

Depending on the application domain and the chosen architecture, a WSN can contain different types of nodes, each with a specific role. In general, we distinguish [10] the following:

- The regular node (RN) which has a transmission unit and a data processing unit;
- The sensor node or source node, which is a regular node, equipped with an acquisition or detection unit;
- The actuator node also called robot which is a regular node which has a unit thanks to which it performs specific tasks (mechanical tasks);
- The gateway node which is a regular node that broadcasts traffic into the network;
- The sink node, which is a regular node with a serial converter connected to a second communication unit. The second communication unit provides a rebroadcast of the data coming from the sensor nodes to a user or to other networks (Internet, for example). It is sometimes called a base station. Table 1 summarizes the types of nodes in a WSN.

However, to optimize key parameters such as network lifetime or data transmission time, some work has focused on WSN architecture. Indeed, the role of each node in the WSN depends on whether the architecture is flat, hierarchical, or multi-level. There are three main roles [10,11]:

- Source Node (SN): Detects phenomena occurring in its immediate environment and broadcasts them either directly or by multiple hops.
- Relay Node (RN): Gathers and relays the measurements coming from the SN. In a flat architecture, an SN can be considered an RN potential. In a 2-level architecture, an RN plays its role for one or more SN. In a 2-tier architecture, the transmission capacity of the RN is assumed to be higher than that of the SN.
- Data Collector Node (DC): In addition to being an RN, it collects measurements from SNs and, potentially, aggregates them. In general, SNs are grouped into several clusters, and one DC is used as the cluster leader for each cluster.

Table 1 summarizes the types of nodes in a wireless sensor network (WSN).

## 3. Types of Deployment in Wireless Sensor Network

The deployment of WSNs occurs in several areas. In [12], the authors propose a multi-UAV-assisted WSN architecture under a Time-Switching Non-Orthogonal Multiple Access (TS-NOMA) protocol, wherein UAVs relay between a central base station, sensor nodes, and D2D pairs to maximize total throughput via joint optimization of relay selection, time allocation, and UAV deployment. UAV-assisted relaying has been shown to extend coverage and capacity in WSNs. Basically, the deployment problem can be defined as follows: given *n* wireless sensor nodes and a target area *A*, to be monitored, determine the optimal placement of these nodes to form a Wireless Sensor Network (WSN) that satisfies specific performance requirements. To address this problem, several deployment strategies have been proposed.

### 3.1. Deterministic Deployment and Stochastic Deployment

A deployment is said to be deterministic when the selection of sensor positions is possible and determined in advance. This approach is used in accessible, non-hostile areas, where sensor nodes can be placed in fixed, known positions according to a predefined plan. Deterministic deployment offers the advantage of optimal network configuration and visibility. However, many WSN applications have to operate in sometimes hostile environments, making deterministic deployment difficult, if not impossible. In such cases, sensors may, for example, be dropped from an aircraft; this is known as non-deterministic or stochastic deployment [7,9,11,13].

### 3.2. Single-Objective Deployment and Multi-Objective Deployment

The criteria and objectives on the basis of which deployment is optimized are often contradictory. In the case of coverage and energy consumption, for example, the better the coverage, the higher the energy consumption [13]. It is difficult to have a deployment that optimizes all objectives simultaneously, although there are methods that try. However, it is usual to find an optimal compromise between the different objectives. Depending on the approach used, it is possible to optimize each objective separately or to use an aggregation function that combines all objectives into a single function, with weights which represent the importance of each objective.

### 3.3. Homogeneous Deployment and Heterogeneous Deployment

The application domain to be deployed will determine whether to deploy sensors of the same type (homogeneous) or of different types (heterogeneous). A WSN is said to be heterogeneous if the distribution of sensors is disparate, or if sensors have different roles, thus characterizing a heterogeneous architecture [11,13]. Different applications require a variety of devices that differ in number, type, and role in the network. Conversely, a wireless sensor network is said to be homogeneous if all of its sensors have the same storage, processing, battery power, sensing, and communication capabilities.

### 3.4. Static Deployment and Dynamic Deployment

By considering node mobility as a criterion, two deployment strategies can be distinguished: the static deployment in which the nodes have unchanged positions and the mobile deployment in which the nodes have an ability to move and reposition after initial deployment [11,13]. In the case of a static deployment, the best node locations are chosen or calculated based on the optimization strategy, and then no position changes will occur during the lifetime of the network. This type of deployment does not take into account the changes (such as the mobility of the targets, for example) during the operation of the network. However, in several use cases, it may be necessary to dynamically reposition the nodes during the operation of the network. The case of moving target coverage, for example. Dynamic deployment is therefore a solution to the problem of the lack of an optimality guarantee that can arise during initial static deployment. It assumes that nodes can move in a coordinated way in the network. However, moving nodes during network operation is often expensive and requires continuous monitoring of network status and events occurring in the vicinity of the node. Table 2 summarizes the types of deployment in a WSN.

Static deployment tends to maximize coverage and minimize energy spent on mobility, making it ideal for protected or infrastructure-powered environments; stochastic (random) deployment sacrifices some coverage guarantee but is indispensable in inaccessible or hostile areas; grid-based deterministic layouts deliver predictable coverage/connectivity with low deployment cost; single- vs. multi-objective formulations trade off between optimizing a single metric (e.g., coverage) or jointly balancing several (e.g., coverage + energy); homogeneous vs. heterogeneous architectures align uniformity against role-specialization; and dynamic (mobile) deployment boosts adaptivity and resilience at the cost of higher control and energy overhead.

For instance, in [14], the authors integrate non-orthogonal multiple access (NOMA) technique and autonomous aerial vehicles (AAVs) in the context of dynamic deployment in WSNs, where mobile relays adjust their positions to maintain coverage and connectivity in time-varying environments. Similar to relay selection [12] and time allocation problems in WSNs, they jointly optimize drone trajectories and spectral resources under QoS and channel uncertainty constraints. Table 2 provides a view and relationship between deployment strategies, performance metrics and ideal scenarios.

## 4. Objectives to Be Met During Deployment

Several objectives can be targeted when sensor nodes are deployed. These objectives are responses to the design constraints that must guarantee an optimal lifetime for the network. In the literature, most deployment methods mainly focus on specific criteria such as coverage, connectivity, overlay, number of nodes, power consumption, and fault tolerance. Depending on the deployment methods, these objectives can be taken into account separately or simultaneously.

### 4.1. Coverage

Ensuring good coverage of the region of interest or targets is considered one of the important metrics in sensor networks. Because of this, coverage in WSN is widely discussed in the literature [9,11,15,16,17,18,19,20,21,22,23,24,25,26,27,28,29,30,31,32,33,34,35,36,37,38,39,40]. In fact, coverage is the real reason for sensor networks. Without it, no phenomenon can be detected and then transmitted for processing. Coverage is therefore the backbone of sensor network applications because it measures the relationship between this network and the environment in which it is implemented.

**Figure 2:**
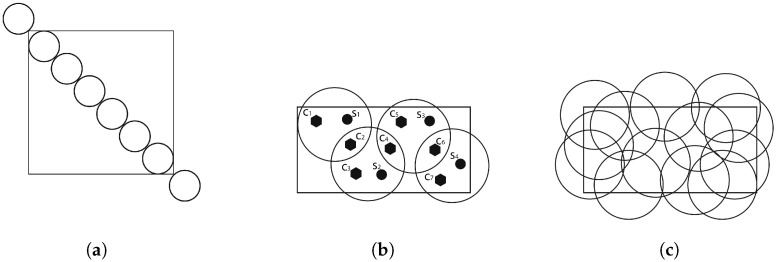
Types of coverage. (**a**) Barrier coverage. (**b**) Target coverage. (**c**) Area coverage.

Coverage reflects how well a sensor monitors a point or area. It is defined as how each point in an area of interest is monitored by a sensor node [23]. Depending on the application for which the network is deployed, different levels of coverage can be implemented. Some applications allow reduced coverage, while others, more critical, require full coverage over the lifetime of the network. There are three types of coverage [7,9,13,21,22,29] illustrated in Figure 2. These are the following:

- Barrier coverage: The objective is to obtain an arrangement of sensors with the task of maximizing the probability of detecting the penetration of a specific target through the barrier. Here, sensors are usually deployed not to detect/track events in an area of interest, but to detect intruders attempting to enter that area. As shown in Figure 2a, the protective barrier formed by the sensors ensures that any movement through it is detected. This type of coverage is suitable for motion or intrusion detection applications.
- Target coverage: The objective is to cover a set of targets whose position is known and which must be monitored. As illustrated by Figure 2b, this coverage scheme focuses on determining the positions of the sensor nodes, guaranteeing effective coverage for a limited number of stationary targets. Each target must be covered by at least one sensor node. The coverage of a target is defined as the number of sensor nodes covering it. As a result, deployment costs are lower because fewer sensors are used compared to the number required to cover the entire area.
- Area coverage: The main purpose is to cover (monitor) a region and maximize the detection rate of a specific area. Depending on the required deployment requirements, full or partial coverage may be requested. However, if the number of sensors is insufficient, complete coverage cannot be achieved, and the objective will therefore be to maximize the coverage rate as shown in Figure 2c.

### 4.2. Connectivity

Connectivity is an important aspect for the network from the point of view of communications between sensor nodes. When a sensor detects an event, it must transmit the information to the base station, also called the sink node. This transmission can be made directly to the sink node or via other nodes of the network.

Since communication between sensors is wireless in a WSN, it is not immediately obvious which other sensor nodes of the network they can communicate with. It is therefore necessary to build the communication graph of the network to have a global view of it. Each sensor node of the WSN constitutes a vertex of this graph. Once built, this graph makes it possible to evaluate the connectivity of the network by highlighting the path(s) going from a sensor node to all the other sensor nodes of the network, including the sink.

**Figure 3:**
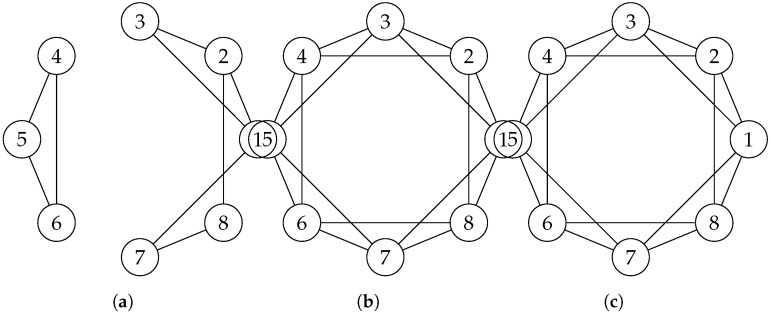
Comparison of connectivity. (**a**) Partial Connectivity. (**b**) 1-connectivity. (**c**) k-connectivity.

The network in a defined area is connected if whatever the pair of sensor nodes, there is at least one path that connects them [30]. Network connectivity is thus defined as the communication capacity between the different nodes of the network where the data are transmitted to the sink by a single or several hops [37]. This communication is conducted via the communication range $r_{c}$. For two sensor nodes to be directly connected, the distance between them must be less than or equal to $r_{c}$. More generally, two sensor nodes are connected if and only if they can communicate directly (one-hop connectivity) or indirectly (multi-hop connectivity) [19]. When a WSN has unique connectivity between sensor nodes, it is called 1-connectivity. The failure of a single node in such a scenario can cause communication failure in the network, splitting it into two. In the case of *k*-connectivity (k>1), despite the failure of a node, the network remains connected by (k−1) number of *n* sensor nodes. Figure 3 shows the three types of connectivity. Partial connectivity, which in most cases corresponds to an unconnected network, 1-connectivity, and the *k*-connectivity with k=2.

### 4.3. Overlap

**Figure 4:**
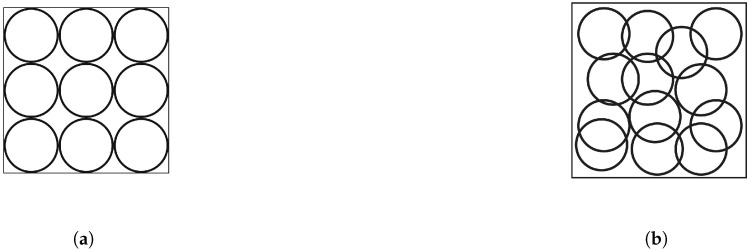
Types of overlap. (**a**) Deployment without overlap of sensor node coverage beams. (**b**) Deployment with overlapping sensor node coverage beams.

Whatever the type of deployment chosen, it frequently happens that the sensor coverage beams, represented here by the circles in Figure 4, overlap.

Some works [24,25,26,28] favor the deployment illustrated by Figure 4a, which excludes overlaps. This type of deployment foresees the juxtaposition of the sensors and, in doing so, ensures maximum coverage of the area of interest, assuming that certain points or areas are without coverage.

However, with this deployment, some goals or constraints may be difficult to achieve. While coverage is provided this way, connectivity is not. Some works, such as [31,32,34,35,36,37], propose deployments, taking into account minimum overlaps. This type of deployment, illustrated in Figure 4b, is more in line with reality and makes it possible to satisfy certain constraints such as connectivity, but can make it more difficult to achieve other objectives, such as maximizing coverage.

It is therefore necessary to find a compromise according to the objectives to be achieved for the deployment and the constraints set by it.

### 4.4. Energy Efficiency

Energy consumption appears in the deployment as a constraint rather than a primary objective. However, it is an important constraint, since sensor nodes are generally equipped with small batteries and become inactive as soon as these batteries run down. Thus, a failed or depleted battery will cause the node to malfunction, which implies topological changes and may require network reorganization. Depending on the application, recharging or changing the energy source may be costly or even impossible due to the deployment environment. To increase the overall lifetime of the network, WSN nodes need to be energy self-sufficient for as long as possible. Energy loss in a sensor node occurs during sensing, communication, and/or data processing [10]. In these processes, communications are generally more expensive than processing [10]. Energy savings are, therefore, mainly achieved through two processes: sensor standby to limit power consumption, and local information processing to avoid losses due to excessive communications. Works such as [34,41,42] focus more specifically on the energy consumption of WSNs, proposing efficient deployments applied to certain application domains.

While energy efficiency techniques focus on reducing the network’s power consumption, energy recovery strategies aim to harvest ambient energy to replenish node batteries and extend operational lifetime. Energy harvesting in Wireless Sensor Networks (WSNs) seeks to extend network lifetime by converting ambient sources—solar, thermal, mechanical vibration, and radio frequency (RF)—into electrical power, each presenting unique trade-offs in power density, reliability, and integration complexity. Solar photovoltaic harvesting offers the highest power yield but is subject to weather variability and requires energy storage management [43]. Thermoelectric generators deliver continuous power in industrial or environmental temperature gradients, though at moderate voltage levels and with reduced efficiency under small temperature differences [44]. Piezoelectric vibration harvesters exploit mechanical oscillations in structures, achieving tens to hundreds of microwatts when tuned to resonance, but demand precise mechanical design and power conditioning circuits [45]. Radio frequency energy harvesting enables battery-less operation in indoor or urban environments by scavenging ambient RF signals, yet its low power availability often necessitates hybridization with other sources [46]. Recognizing these limitations, recent work advocates hybrid energy harvesting systems that combine multiple transduction methods to ensure reliable, continuous power, exemplified by strategies for integrating supercapacitor-based, battery-less harvesters into multi-hop industrial WSNs [47]. Table 3 provides a comparison of energy harvesting strategies in WSN.

### 4.5. Node Synchronization

Node synchronization underpins coordinated functions in wireless sensor networks (WSNs)—from data fusion and duty cycling to medium access control—but must do so with minimal energy use and communication overhead. To this end, recent work has explored architectures and protocols that balance precision with efficiency. For example, Wang et al. propose FLTS, a mesh-star design that partitions the network into a routing layer and a sensing layer, thereby speeding convergence and cutting messaging traffic [48]. Building on this layered approach, Liu et al. introduce a pulse-based scheme that uses neighbor-to-neighbor exchanges to eliminate propagation delays altogether, achieving high accuracy without a global reference clock [49]. Recognizing that multi-hop networks still accumulate errors, Shi et al., in [50], develop a rapid-flooding protocol with real-time delay compensation to curb per-hop drift in large deployments. To cover diverse application needs, Hababeh et al. integrate multiple synchronization strategies into a unified framework that can be tuned for cost, reliability, hierarchy, and security [51]. Finally, Chen et al. enhance industrial WSNs with a reference-broadcast protocol hardened against Sybil attacks and message tampering, marrying precision timing with robust authentication [52]. Table 3 presents a comparison of energy harvesting strategies in WSNs.

### 4.6. Localization Techniques

Accurate localization in wireless sensor networks (WSNs) demands a balance between precision, cost, and complexity. Ultra-wideband (UWB) Time Difference of Arrival (TDoA) methods achieve sub-meter accuracy by exploiting large bandwidth and fine time resolution; however, they require specialized hardware and precise time synchronization, making them best suited for indoor or controlled environments. To reduce infrastructure demands, Fawad et al. enhance the classic DV-Hop algorithm with optimized hop-count correction and energy-aware filtering, yielding localization errors below 2 m while preserving low computational overhead [53]. Building on hybrid paradigms, Wang et al. integrate a chaotic porcupine optimizer into a DV-Hop framework, which adaptively tunes hop-distance estimates to achieve high reliability in agricultural deployments with minimal anchor nodes [54]. Together, these approaches illustrate a trend toward hybrid and learning-based localization schemes that deliver robust accuracy with manageable hardware and energy costs.

### 4.7. Fault Tolerance

In any type of network, malfunctions of any kind can occur. Bad weather, hardware problems, or excessive energy consumption, for example, can render a sensor node inactive. This creates a malfunction that can be critical if action is not taken beforehand. The WSN must therefore be fault-tolerant. In this respect, fault-tolerant deployment strategies are used to prevent individual failures that reduce the overall lifetime of the network. Examples include load balancing between nodes, deploying new nodes and relocating them, etc. The work in [55] presents a survey of existing work from 2002 to 2019, followed by an overview of existing fault tolerance protocols with a comparative analysis.

### 4.8. Number of Nodes

The criterion of the number of nodes is almost always a financial constraint, and sometimes an energy or environmental one. The number of nodes to be deployed is an important question prior to any deployment, as it is closely linked to the application and WSN objectives. Depending on the application, the number of sensor nodes required may vary. For example, the same number and type of nodes will not be deployed for a home automation application, a city traffic monitoring application, or an agricultural application. In addition to the WSN application, the objectives to be achieved are also a determining factor in calculating the number. Depending on whether the objective is coverage or connectivity, or both, or the need to save sensor energy, the number of sensors can significantly affect network efficiency.

## 5. Detection Models

Whatever the type of coverage desired during deployment, it is deduced from the coverage associated with each of the sensors that form the network. A general fading model may not always best mitigate the real phenomenon of fading in case of diverse deployment scenarios. It is therefore necessary to have a model of the coverage of a sensor in order to express the total coverage of the network. This model is based on the detection range (or radius) ($r_{s}$) of the sensor and is often simplified compared to reality. Generally, two main hedging models recur: the deterministic or binary model and the probabilistic model [9,10,11,13,15].

### 5.1. Binary Model

The deterministic model is the simplest and is widely used [13,15,24,25,26,27,33]. In this model, the detection range of each node is illustrated by a disc of radius $r_{s}$ called the radius or detection range. It is based on the idea that all points located in a disk centered on the sensor at location $\left(x_{s},y_{s}\right)$ are supposed to be covered by it. The center of the circle therefore indicates the position of the sensor and its radius, its detection range. Thus, a sensor node only detects events that are within its detection range. Any area or point that is outside this detection range is not monitored. The coverage function used is defined by Equation (1):

(1) $$f\left(s,p\right)=\left\{\begin{array}{cc} 1 & \;\mathrm{if}\;d\left(s,p\right)\leq r_{s}\\ 0 & \;\mathrm{else}. \end{array}\right.$$

where d(s,p), the Euclidean distance between the sensor *s* and a point *p*, is given by Equation (2):

(2) $$d\left(s,p\right)=\sqrt{{\left(x_{s}-x_{p}\right)}^{2}+{\left(y_{s}-y_{p}\right)}^{2}}$$

### 5.2. Probabilistic Model

Considered simplistic, the binary detection model does not take into account the measurement uncertainty factor. This is why some works use the probabilistic model. The probabilistic detection model in WSNs aims to determine the probability of an event occurring based on sensor measurements and takes into account various factors that may affect these measurements. There are several types of probabilistic models, such as Elves, Shadow Fading, Rayleigh Fading, and Nakagami-m Fading [15].

#### 5.2.1. The Elves Model

In this model, the detection behavior of sensor nodes is more uncertain. The distance that separates the target to be monitored from the node and the characteristics of the node itself influence the perception of the node and its ability to detect the target [10,13,56]. The detection capability of the sensor node decreases as the distance between the node and the event increases. This model reveals the performance of detection range devices and is affected by various factors like noise, obstacles, and interference [9,11,15,33,56]. This makes it more realistic and more consistent with real-world deployments.

In the Elves model, for a sensor *n*, two distances are defined. The first distance $r_{s}$ defines a detection distance where the target is detected with a probability of 1 if the distance between the target and the node is less than $r_{s}$. The second distance, $r_{i}$ represents an uncertain detection distance, such that $r_{i}$ is smaller than $r_{s}$. The expressions $r_{s}$−$r_{i}$ and $r_{s}+r_{i}$ define a ring in which an event may or may not be detected depending on the value of the coverage probability. A node could then detect with a probability *p* a point or an object located in the interval between $r_{s}-r_{i}$ and $r_{s}+r_{i}$. The probability of coverage of a point P(x,y) by the sensor *n* is then given by Equation (3):

(3) $$C_{xy}\left(n\right)=\left\{\begin{array}{cc} 0 & \;\mathrm{if}\;r_{s}+r_{i}\leq d\left(n,P\right)\\ e^{-\lambda \alpha^{\beta }} & \;\mathrm{if}\;r_{s}<d\left(n,P\right)<r_{s}+r_{i}\\ 1 & \;\mathrm{if}\;r_{s}\geq d\left(n,P\right) \end{array}\right.$$

where λ and β are constants depending on the characteristics of the sensor node and α is given by Equation (4):

(4) $$\alpha =d\left(n,P\right)-\left(r_{s}-r_{i}\right)$$

#### 5.2.2. The Shadow Fading Model

Also known as log-normal shadowing, this model refers to the variation in signal strength caused by obstacles and obstructions in the propagation path of wireless signals [15,57]. As mentioned above, these obstacles can include buildings, trees, terrain features, or other objects that block or reflect the signal [40]. Shadow fading is a random phenomenon that follows a log-normal distribution, which means that the signal strength at a particular location can be modeled as a random variable with a specific mean and variance. The model is used to account for the uncertainty introduced by the random variations in signal strength due to shadow fading. By considering the Shadow Fading model, the detection algorithm can estimate the likelihood of an event based on the observed sensor measurements and the expected variations caused by shadow fading.

By using the shadow fading model, the WSN can improve the accuracy and reliability of event detection by accounting for the variations introduced by shadow fading. This helps to mitigate false positives or false negatives in the detection process, leading to more robust and efficient intrusion detection in WSNs, for instance. The shadow fading model is typically expressed using a log-normal distribution, which is a probability distribution that describes the random variations in signal strength due to shadow fading [15]. The probability density function of the log-normal distribution can be expressed as in Equation (5):

(5) $$f\left(x\right)=\frac{1}{x\sigma \sqrt{2\pi }}e^{-\frac{{\left(\ln\left(x\right)-\mu \right)}^{2}}{2\sigma^{2}}}$$

where

- *x* is the random variable representing the signal strength;
- μ is the mean of the logarithm of the signal strength (in decibels),
- σ is the standard deviation of the logarithm of the signal strength (in decibels);
- π is a mathematical constant approximately equal to 3.14159.

This probability density function gives the probability of observing a specific value of the random variable *x*. To calculate the probability of an event occurring given a threshold value, it will be necessary to integrate the probability density function over the desired range. Additionally, the specific values for μ and σ should be determined by empirical measurements or by using channel modeling techniques specific to the wireless environment in which the WSN is deployed. These values may vary depending on factors such as the characteristics of the environment and the frequency of the wireless signal used.

#### 5.2.3. The Rayleigh Fading Model

The Rayleigh fading model is a commonly used statistical model to describe the variations in signal strength in wireless communication systems [15,42]. It is particularly applicable in scenarios where there are multiple uncorrelated signal paths between the transmitter and the receiver, resulting in a phenomenon known as multi-path propagation [15,42,57]. In the Rayleigh fading model, the signal strength is considered to be a random variable that follows a Rayleigh distribution. This distribution characterizes the amplitude of a signal after passing through multiple independent paths with different delays and attenuation. It is a statistical model that assumes no dominant path or line-of-sight component in the signal propagation.

The probability density function of the Rayleigh distribution is given by Equation (6):

(6) $$f\left(x\right)=\frac{x}{\sigma^{2}}\;\;e^{\frac{-x^{2}}{2\sigma^{2}}}$$

where

- *x* is the random variable representing the amplitude of the signal;
- σ is a scale parameter that determines the spread of the distribution.

Similar to the log-normal distribution, the Rayleigh fading model is used to capture the statistical variations in the received signal strength due to multi-path propagation. The model assumes that the magnitude of the received signal can vary around a mean value of zero, and the variations follow the Rayleigh distribution [15]. In practical implementations, the Rayleigh fading model is often combined with other models to capture additional factors that affect signal propagation in WSNs. By incorporating the Rayleigh fading model into the probabilistic model for detection, the WSN can account for the random fluctuations in signal strength due to multi-path fading, improving the accuracy of event detection and overall system performance.

#### 5.2.4. The Nakagami-m Fading Model

Also commonly used to describe the variations in signal strength in wireless communication systems, this model is often used as an alternative to the Rayleigh fading model and provides a more generalized representation of fading phenomena [15]. The signal strength is modeled as a random variable that follows a Nakagami distribution. This distribution characterizes the amplitude of a signal after passing through a wireless channel with a varying number of independent paths. The Nakagami-m distribution can exhibit a range of fading characteristics, from severe fading (m<1) to mild fading (m>1) [58]. The probability density function of the Nakagami-m distribution is given by Equation (7):

(7) $$f\left(x\right)=\frac{2m^{m}}{\Gamma (m)}\;\;x^{2m-1}\;e^{\frac{-mx^{2}}{\Omega^{2m}}}$$

where

- *x* is the random variable representing the amplitude of the signal;
- m is the fading parameter that determines the severity of fading (0≺m≤∞);
- Γ(m) is the gamma function;
- Ω is the average power of the received signal.

The fading parameter *m* influences the shape and characteristics of the Nakagami-m distribution. As *m* approaches infinity, the distribution approaches a Gaussian distribution, representing less severe fading, whereas for smaller values of *m*, the distribution exhibits more pronounced fading effects [15,58]. The Nakagami-m fading model is commonly used in scenarios where there are both line-of-sight (LOS) and non-line-of-sight (NLOS) signal components. It provides a more flexible and versatile representation of fading compared to the Rayleigh fading model, which assumes no dominant path. By adjusting the fading parameter *m*, the model can capture a wide range of fading conditions and accurately simulate the variations in signal strength in WSNs [15]. Table 4 presents different detection models in WSN.

In practical implementations, the value of *m* is typically determined based on empirical measurements or channel modeling techniques specific to the wireless environment. By incorporating the Nakagami-m fading model into the probabilistic model for detection, the WSN can account for the varying severity of fading and improve the accuracy of event detection and system performance in realistic wireless environments.

## 6. Methodologies for Deploying

There are several methods for deploying sensors. These are mainly classic deployment methods and metaheuristics [9]. Some works add a third category of methods based on self-planning techniques [33]. Classical deployment methods fall into three broad categories: Grid-Based Techniques, Force-Based Techniques, and those on Computational Geometry [9,33].

### 6.1. Grid-Based Techniques

Grid-based strategy is a type of deterministic deployment where the positions of sensor nodes are fixed according to a regular grid pattern, such as a triangular grid, a square grid, or a hexagonal grid. According to the deployment algorithm that has been commonly used, the monitoring area is divided into small grids and the sensor nodes are located in the center or at the vertices of the grid. The regular deployment pattern of sensor nodes in the target area can be considered an appropriate solution to provide an acceptable degree of coverage and connectivity with a minimum number of nodes. Optimal deployment patterns can be obtained based on the relationship between $r_{s}$ and $r_{c}$ [9,33].

The authors of [17] provide a solution to find the optimal deployment to have *p*-*coverage* and *q*-*connectivity*, where *q* is less than or equal to 6 for some typical values of $\frac{r_{c}}{r_{s}}$. In this work, for different ratios between $r_{s}$ and $r_{c}$, the authors proposed three different shapes, namely a triangular lattice, a square lattice, and a hexagonal pattern [9,33]. They compare the number of sensor nodes needed for regular deployment patterns to achieve coverage of 1, 3, and 5 and connectivity of *q* with $r_{s}=30$ m using Equation (8):

(8) $$N=\frac{area\;to\;cover}{T_{a},S_{a}\;or\;H_{a}}$$

where *N* is the number of nodes, $T_{a}$ is the triangular surface, $S_{a}$ is the square surface, and $H_{a}$ the hexagonal surface. The authors of this work recommend the triangular lattice model as the appropriate deployment model in case the required coverage level is greater than or equal to 3, with the ratio $r_{c}/r_{s}\geq \sqrt{3}$ [33].

In [18], the authors introduce a coverage algorithm based on a virtual square grid, referred to as the Virtual Square Grid-based Coverage Algorithm (VSGCA). In this approach, each sensor node partitions its sensing area into virtual square grids. A node is considered redundant if all its associated grids are already covered by neighboring nodes. Compared to several existing algorithms, VSGCA ensures both full coverage and network connectivity over the entire area of interest, while achieving a low spatial complexity of O(n+M) and a time complexity of O(n×M), which is lower than that of most competing algorithms, with *n* representing the number of neighboring nodes and *M* the total number of grid points for each sensor node [17,18]. However, this algorithm does not take into account the scenario of obstacles and the mobility of nodes.

In [56], the authors propose an improved grid-based joint routing and charging algorithm (Improved grid-based joint routing and charging algorithm—IGRC). To balance the energy consumption of the network, a mobile charger starts from the base station located in the center of the network and then uses the nearest neighbor method to visit the nodes of the three interior square rings. It moves along the edge of the outer rings and stops at each vertex to charge the nodes of the outer rings. For each square ring, the time spent on a given load point is determined by the average power consumption rate of the nodes located in the ring. The charging time allocated to each square ring can be calculated accurately based on different power consumption rates. The IGRC ensures that each node can replenish its energy before falling below a critical threshold.

### 6.2. Techniques Based on Virtual Force

The Virtual Force Algorithm (VFA) is one of the most popular mechanisms to solve the problem of node coverage and deployment in sensor network applications wireless. In this mechanism, the sensors move thanks to the virtual force determined by the relative position of the neighboring nodes and the presence of an event [16].

The virtual physical model strategy considers the sensor node as an electron or a molecule and uses the idea of virtual forces, which can be repulsive, attractive, or null [9,13,16,33]. These forces act on the pair of adjacent sensor nodes depending on their distance from each other. If the distance between two adjacent sensor nodes is greater than a pre-calculated threshold value, the attractive force will be exerted; likewise, if this distance is less than the predefined threshold value, the force that will be exerted will be of a repulsive nature, and finally, if the distance is equal to the threshold value, the null force will be exerted. However, their overall network topology is relatively dense in the center and sparse at the edges due to physical reasons. In this strategy, it is assumed that all the sensors have identical detection, communication, calculation, and mobility capabilities. A sensor can communicate with any other sensor within the $r_{c}$ communication range, and they can monitor the region and collect data within the $r_{s}$ detection range. If the sensor network was deployed in the perfect hexagon topology, the distance *d* between two neighbors is $\sqrt{3}\times r_{s}$. To therefore communicate with neighboring nodes, the communication range of each sensor must be greater than [16].

In order to effectively improve the coverage of a WSN in the surveillance area, the authors of [56] proposed a coverage optimization algorithm for wireless sensor networks with a Virtual Force algorithm -Lévy-embedded Gray Wolf optimization (VFLGWO). In practice, they apply the Virtual Force to the Lévy-embedded GWO (LGWO) algorithm, in order to have the VFLGWO algorithm. This algorithm allows them to obtain a higher coverage rate of the monitoring area, a more uniform distribution of sensor nodes, and a shorter average distance of movement along the wireless sensor nodes.

While a complex strategy to implement, VFA provides adequate coverage for the viewing area while maintaining network connectivity and coping with obstacles in the viewing area. However, in this strategy, the sink node requires a high computational capacity, due to the computation of the new positions. Then, VFA performance is not good when dealing with a heterogeneous RCSF, due to virtual forces, and finally, this technique only applies to mobile nodes. To assess the uniformity of the node distribution, the VFA uses an assessment metric called the pairwise correlation function.

### 6.3. Techniques Based on Computational Geometry

The computational geometry strategy is based on geometric objects such as points, polygons, and line segments [33]. As an example, computational geometry is used to solve the famous art gallery problem, where the entire gallery boundary is guarded by at least one of the security guards. To solve this problem, one needs to estimate the minimum number of cameras that can be placed in a polygonal environment in order to monitor each point of the environment. Most calculation techniques rely on irregular patterns, which are more complex than regular patterns.

Two of the most popular basic algorithms are Delaunay triangulation and Voronoi diagram [9,33], both stemming from computational geometry methods used in RCSF. The Voronoi diagram is a method of partitioning the area of interest into a number of Voronoi polygons based on the distances between the sensor nodes [9,33].

Each node occupies only one polygon, all interior points of which are closer to it than to any other node. On the other hand, the Delaunay triangulation is the dual graph of the Voronoi diagram. It is constructed by connecting any two adjacent points of the Voronoi diagram whose polygons share a common edge. Most of the time, Voronoi and Delaunay triangulation diagrams are used to eliminate or at least reduce coverage hole problems in RCSFs.

There are also very handy algorithms, such as Voronoi-based algorithms (VOR), Vector-based algorithms (VEC) [9,33], and Mini-max [33] that allow for the discovery of difficulties related to coverage holes. The VEC algorithm is taken from the behavior of electromagnetic particles, and it nudges the sensor nodes away from the crowded covered area. Two sensors exert a repulsive force when the distance separating two nodes is small. As for the VOR, it removes the sensor nodes from the sparsely covered area. Finally, the Mini-max algorithm is similar to the VOR in that it reduces coverage holes by bringing sensor nodes closer to the farthest Voronoi vertex. Mini-max chooses the position of the target node as the point inside the Voronoi polygon that minimizes the distance to the furthest Voronoi vertex [33]. The most important advantage of these three algorithms is that it is a distributed approach that can help the network scale to a large scale and reach. They also ensure connectivity is maintained while reducing coverage gaps. In addition, they have the ability to deal with obstacles in the monitoring area. On the other hand, the complexity of detecting coverage holes and estimating new node positions is costly. Additionally, they have poor performance on initial cluster deployment and lower communication range.

### 6.4. Heuristic Optimization

In addition to mathematical programming, heuristics are exclusively algorithmic solution methods that are used to quickly obtain solutions to any decision-making problem. In some cases, the use of mathematical programming methods can be very complex and therefore add a very long calculation time.

A heuristic is an approximate method of solving an optimization problem. It is an algorithm that aims to find a feasible solution while respecting a set of constraints and criteria without guaranteeing optimally, but within a reasonable resolution time [9,13,17,59]. It consists of a common-sense strategy to move intelligently in the space of solutions, to have an approximate solution, the best there is, in a reasonable time [59].

There are two types of heuristics which are generally used: those of construction (greedy methods, for example), which build in an iterative way a solution, and the heuristics of descent, which, starting from a fixed solution, seek a local optimum. The heuristics used depend on the problem to be solved, especially in the choice of neighborhood. The heuristic solution is considered by a set of decision variables describing a system, for example. These variables are defined on continuous or discrete domains [13]. If it is a discrete domain, we speak of combinatorial optimization and the set of solutions is countable. On the other hand, if certain variables are linked to each other, we speak of continuous optimization.

More advanced heuristics were created and resulted in a new family of algorithms called [59,60] metaheuristics. The goal of a metaheuristic is to succeed in finding a global optimum. To achieve this, they traverse the search space while exploring areas that seem promising, taking care not to be trapped in a local optimum. Metaheuristics are generally the example of natural processes and are increasingly used with other optimization methods.

The main metaheuristics [59] for solving problems with discrete variables are as follows:

- The simulated annealing, which will traverse the search space while allowing itself to deteriorate its solution to leave the local optima. During the process, the annealing will accept less and less of these deteriorations, which will make it converge towards an optimum, which is hoped to be global [60].
- Search with taboos, unlike simulated annealing, is deterministic and has a notion of memory. The selection of the best neighbor of a solution leads the algorithm to detect the local optima, and since the examination of the search space is conducted by limiting the neighborhood of the solution by making certain moves “taboo”, the algorithm must theoretically examine the global optimum [60]. In [61], the authors proposed a low-power multi-hop adaptive clustering hierarchy protocol. In their work, they propose an optimization of the head of a cluster in the RCSFs by working on the selection of the optimal path in the routing so as to improve the lifetime as well as the energy efficiency of the network. To obtain better results, they used, in addition to the method of optimization by swarms of particles, the research with taboos. This allowed them to avoid the problems of poor local optima that can be encountered using only certain metaheuristic techniques, in particular, optimization by particle swarms. By doing so, they manage to improve the number of clusters formed, the percentage of alive nodes and the reduction in the average packet loss rate and the average end-to-end delay in the RCSF.
- Evolutionary algorithms, resulting from Darwin’s theory of evolution, manipulate several solutions at the same time by combining them to form new solutions. Having a population of solutions simplifies the examination of the search space. The best solutions will be chosen to participate in the creation of new solutions, which will favor the combinations of “good characteristics”, and will make it possible to find a global optimum [60]. Several works [24,25,26,27,28,29,31,62] use, for example, genetic algorithms in the deployment of RCSFs to achieve different objectives. In these works, the authors generally propose an optimal compromise between the coverage, the connectivity, and the lifetime of the network. While the authors of [24,25] worked on how to maximize area coverage in the deployment of homogeneous wireless sensor networks based on an efficient genetic algorithm, the authors of [27], on the other hand, focused on a deployment seeking to maximize target coverage. In [31], the main objective of the authors was to present a dynamic deployment technique based on a genetic algorithm in order to maximize the area coverage with the smallest number of nodes while minimizing overlaps between neighboring nodes. In [35], the authors proposed a genetic algorithm approach for placement of *k*-coverage and *m*-connected nodes in target-based wireless sensor networks. Given a set of target points, they found the minimum number of potential positions to place the sensor nodes respecting both coverage and connectivity by proposing a scheme based on a genetic algorithm.

## 7. Open Issues and Future Research Directions in WSNs

Despite significant advancements in Wireless Sensor Network (WSN) deployment strategies, several open issues persist, necessitating further research to enhance efficiency, scalability, and adaptability. One critical challenge lies in optimizing node placement to balance coverage, connectivity, and energy consumption, especially in large-scale deployments. Recent studies have explored multi-objective optimization techniques to address these conflicting goals, yet achieving an optimal balance remains elusive [63,64].

The integration of machine learning, particularly deep learning models like Stacked Autoencoders combined with Probabilistic Neural Networks, has shown promise in predicting optimal deployment configurations. However, these approaches often require substantial computational resources and large datasets, which may not be feasible in all deployment scenarios [65].

Three-dimensional (3D) deployment introduces additional complexity, as it must account for varying elevations and obstacles in environments such as multi-story buildings or urban canyons. Recent research has begun to address 3D deployment optimization, but comprehensive solutions that consider real-world constraints are still under development [63].

Another area requiring attention is the deployment of WSNs in dynamic and heterogeneous environments. The use of mobile sensor nodes and the need for real-time adaptability pose challenges for maintaining network stability and performance. Studies have proposed various strategies for dynamic deployment, yet practical implementations are limited [66,67].

Furthermore, the deployment of WSNs in complex structures, such as multi-story buildings, demands tailored strategies to ensure effective monitoring and data collection. Research has highlighted the importance of considering architectural nuances in deployment planning, but standardized methodologies are lacking [68].

Lastly, the emergence of virtualization [69,70] in WSNs presents opportunities for resource sharing and improved scalability. However, the implementation of virtual sensor networks introduces challenges related to synchronization, data integrity, and system complexity that warrant further investigation [71].

Addressing these open issues requires a multidisciplinary approach, combining advancements in optimization algorithms, machine learning, and system design to develop robust, adaptable, and efficient WSN deployment strategies.

## 8. Conclusions

This comprehensive review has explored the complex landscape of Wireless Sensor Networks (WSNs) deployment strategies, shedding light on the myriad of considerations, methodologies, and goals that underlie the optimal establishment of these networks. Throughout this exploration, it became evident that the deployment phase is a crucial moment, influencing the overall efficiency, reliability, and adaptability of the network to the intended application domain. It therefore requires a holistic approach.

Understanding the different types of sensor nodes, their capabilities, and their limitations provides the foundation upon which effective deployment strategies can be built. The research and advances discussed in this review demonstrate the complexity and interdisciplinary nature of WSN deployment methodologies. As technology evolves, new challenges arise. Thus, by carefully selecting deployment strategies tailored to application requirements, researchers and practitioners can harness the power of sensor networks to gain meaningful insights into their environment. As the world continues to embrace the Internet of Things (IoT) and the seamless interconnection of devices, the insights provided by this study serve as a road map for orchestrating the successful deployment of wireless sensor networks in a multitude of areas. Ultimately, the deployment strategies discussed here lay the foundation for a smarter, more interconnected future.

## Figures and Tables

**Table 1:** Types of nodes in a wireless sensor network (WSN).

Node Type	Core Components	Roles and Responsibilities	Communication Interface and Range	Energy Source	Processing Capacity
Regular Node	Radio transceiver, microcontroller, memory, power unit	Packet forwarding, basic routing, neighbor discovery	Low-power radio (e.g., IEEE 802.15.4), 10–100 m	Battery (Li-ion, alkaline) or ambient energy harvesting	MCU 8–32 MHz, 16–256 KB RAM
Sensor Node	Regular node components + sensing unit(s), A/D converter	Periodic or event-driven data acquisition, local filtering/aggregation	Same radio + I²C/SPI interfaces for external sensors	As Regular Node	MCU with sensor driver support, moderate on-board storage
Actuator Node	Regular node + D/A drivers, relays, motor drivers	Executes physical commands (motions, switching)	Same radio interface as Sensor Node	Higher-capacity battery, supercapacitors	MCU with real-time OS (TinyOS/Contiki)
Gateway Node	High-power radio (Wi-Fi, cellular), Ethernet/Wi-Fi module, powerful CPU, extended memory	Data aggregation, protocol translation, network management, remote updates	Multi-interface: IEEE 802.15.4 (WSN) + Ethernet/Wi-Fi/LTE (backhaul)	Mains power (redundant supply)	CPU > 100 MHz, > 1 MB RAM
Sink Node	High-power transceiver, high-performance CPU, large storage, serial converter	Final data collection, data fusion/analysis, cloud/server interface	Same as Gateway + optional satellite/mesh backhaul	Mains or UPS backup	Multi-core CPU, gigabytes of RAM/storage

**Table 2:** Deployment strategies vs. performance metrics and ideal scenarios.

Strategy	Key Metrics	Ideal Scenarios
Static/Deterministic	Coverage ↑, Connectivity ↑, Mobility energy ↓	Fixed-infrastructure sites; factory floors; smart buildings where nodes can be placed precisely.
Stochastic/Random	Coverage variance moderate, Connectivity probabilistic, Low planning cost	Disaster zones, battlefields, environmental drops (aircraft); rough terrain where manual placement is infeasible.
Grid-based (Triangular/Square/Hexagonal)	Uniform coverage, Predictable connectivity, Node count optimal	Agricultural monitoring, large-scale environmental surveys, perimeter surveillance requiring regular spacing.
Single-Objective Optimization	Maximizes one metric (e.g., coverage or lifetime), Simpler computation	Applications with a hard primary goal (e.g., gas leak detection demands maximal sensing).
Multi-Objective Optimization	Balanced coverage/energy/latency, Compromise solutions	Quality-critical WSNs (e.g., structural health monitoring) requiring trade-offs between lifetime and QoS.
Homogeneous	Uniformity, Easier management, Lower deployment cost	Low-cost mass deployments (e.g., temperature logging) where identical nodes suffice.
Heterogeneous	Specialized roles (sensors, actuators, relays), Enhanced functionality	Industrial automation, smart grids, or precision agriculture needing both sensing and actuation.
Dynamic/Mobile	Adaptivity, Fault tolerance, Coverage healing	Vehicular networks, emergency response, mobile target tracking requiring repositioning.

**Table 3:** Comparison of energy harvesting strategies in WSNs.

Source	Power Density	Advantages	Challenges	Ref.
Solar	High (mW–W/cm^2^)	High yield, mature technology	Weather-dependent, storage overhead	[43]
Thermal (TEG)	Moderate (µW–mW/cm^2^)	Continuous in gradients	Low voltage, efficiency drops at small ΔT	[44]
Piezoelectric	Variable (µW–mW)	Suitable for structural/vibration contexts	Resonance tuning, narrow bandwidth	[45]
RF	Low (µW–µW/cm^2^)	Ubiquitous indoor sources	Low energy density, rectenna design	[46]
Hybrid Systems	Combined	Enhanced reliability, seamless switching	Increased complexity, cost	[47]

**Table 4:** Detection Models in WSNs.

Model	Description	Consideration	Mathematical Expression
Binary	Simplest model based on circular detection range.	Assumes all points within the sensor’s range are covered.	$f\left(s,p\right)=\left\{\begin{array}{cc} 1 & \mathrm{if}\;d\left(s,p\right)\leq r_{s}\\ 0 & \mathrm{else} \end{array}\right.$
Elves	Probabilistic model considering uncertain detection distances.	Accounts for node distance and characteristics.	$C_{xy}\left(n\right)=\left\{\begin{array}{cc} 0 & \mathrm{if}\;r_{s}+r_{i}\leq d\left(n,P\right)\\ e^{-\lambda \alpha^{\beta }} & \mathrm{if}\;r_{s}<d\left(n,P\right)<r_{s}+r_{i}\\ 1 & \mathrm{if}\;r_{s}\geq d\left(n,P\right) \end{array}\right.$
Shadow Fading	Accounts for signal strength variations due to obstacles.	Models signal strength variations using log-normal distribution.	$f\left(x\right)=\frac{1}{x\sigma \sqrt{2\pi }}e^{-\frac{{\left(\ln\left(x\right)-\mu \right)}^{2}}{2\sigma^{2}}}$
Rayleigh Fading	Models signal strength variations due to multi-path propagation.	Follows Rayleigh distribution for random amplitude variations.	$f\left(x\right)=\frac{x}{\sigma^{2}}e^{-\frac{x^{2}}{2\sigma^{2}}}$
Nakagami-m Fading	Generalized fading model for LOS and NLOS scenarios.	Characterized by Nakagami distribution with fading parameter *m*.	$f\left(x\right)=\frac{2m^{m}}{\Gamma (m)}x^{2m-1}e^{-\frac{mx^{2}}{\Omega^{2m}}}$

## Data Availability

Data supporting reported results can be found with authors, including links to publicly archived data-sets analyzed or generated during the study.
